# Prof. T. F. Rochester’s Medical Clinic at the Buffalo General Hospital

**Published:** 1881-03

**Authors:** Frederick Peterson


### PROF. T. F. ROCHESTER'S MEDICAL CLINIC AT THE BUFFALO GENERAL HOSPITAL.

REPORTED BY FREDERICK PETERSON, M. D.

### JANUARY 26, 1881.

CASE I.—E. W.; aged 17; Canadian; milk vender; has incontinence of urine; nocturnal in character. This affection is rare in boys of his age, but common in little children. It is more frequent with boys than girls, beginning generally at about the second year, ceasing spontaneously at the eighth, ninth or tenth year. In most cases the urine should be examined. There may be an irritating deposit of phosphates. Ascarides, constipation of the bowels, and elongation of the prepuce, requiring circumcision, are frequent causes. Patients with incontinence should eat and drink little in the evening, and at eleven o'clock should be awakened to urinate. Dr. Rochester often adopts the mechanical device of an old physician of this city, of fastening a ball, by means of a belt, to the back of the child, because the micturition generally occurs when the patient is on his back. The medicinal agents most in vogue are mild diuretics, acetate or citrate of potash, when there is a deposit, cubebs and ergot; a rectal injection of 5—10 gr. Chloral Hydrate at bedtime is sometimes efficacious. Masturbation must not be overlooked as a cause of incontinence. This patient is feeble and weak, but has good appetite, and bowels are regular. The following treatment will be used to him: Fl. ex. ergot ʒi every night, and Tr. Ferri Mx t. i. d., and the mechanical expedient if necessary.

Case II.—X. S.; aged 52; laborer; ill four days; temperature 97½°; pulse 96; supposed to be pneumonia, but the low temperature is against it. The temperature in pneumonia ranges from 102—106°. It may be consolidation of the lung. Is affected with prurigo senilis, a nodular skin disease, occurring in winter in old people, and generally dependent upon hepatic disease or nervous disorder. His breathing is very rapid and abdominal. There is epigastric fulness, no vomiting. The swelling in the epigastric region is like that of a tumor; there is pain and dullness, due either to liver or pancreas. Has dyspnœa and rusty expectoration. Sonorous rāles are heard all over the chest, but more in left side; respiration is 42; there is no respiratory murmur in lowest lobe of right lung; a rāle like a dove-coo over left nipple; heart tumultuous, and sounds masked by those of lung. Urine is loaded with phosphates. Believes him to have hepatic and lung disease. A few days will decide. Recommends am. carb. gr. x every 2 h., whisky ℥ss. e. hr., beeftea, a poultice to chest, and three Pil. Cath. Comp.

The Professor then exhibited some post mortem specimens sent to him by Dr. Folwell. The history of the case was as follows: Male; aged 37; book-keeper; 8 months before had indications of hepatitis; there was hepatic enlargement and jaundice. There was also great dyspnœa at times. Upon examination, the heart was found hypertrophied, and with a systolic murmur in mitral region. Dropsy came on. There was no albumen in the urine. The patient improved in health under proper treatment. Last Thursday he was out sleighing, and Friday, all day and evening, was in reasonably good health and spirits. He retired at 10 P. M., and at 3 A. M. was found dead. Upon post mortem examination the liver showed evidences of hepatitis, was softened and contracted; the lungs were healthy; there were remains of an old pleurisy; the kidneys were hypertrophied and engorged, the right one especially; the left kidney was granular; there was no fluid in the pricardium; the heart is typical of hypertrophy; there is no apex—it is rounded, right auricle enormously distended with blood, weighs 20 oz.; the cavity of the left ventricle is twice its usual size, walls thick; the aorta and pulmonary valves healthy; the mitral valve is insufficient, shortened and thickened; has seen one such valve before; it looks as if a cresentic piece had been clipped out. The man died of heart clot, which is enormous. This used to be called "polypus of the heart." Cholera patients often die suddenly from this cause. In the tricuspid valves we find also ulcerative disease. Such cases have been called apoplexy.

FEBRUARY 2, 1881.

CASE I.—The same as No. 2 of last week; apparently little changed since his first appearance; expectorates blood; is somewhat more emaciated than before; respiration still 42; on examination of heart find apex beat in sixth intercostal space, and a weak regurgitant systolic murmur. There is hypertrophy with dilatation and mitral lesion. There is also a very faint murmur over aorta. His urine is 5 per cent. albumen. He probably has triple lesion, cirrhotic liver, renal and cardiac disease. The blood-globules of the expectoration, if examined under the microscope, will be found stellate and shrunken; the legs are œdematous and the circulation so feeble that senile gangrene has commenced. This will gradually extend and patient will die. When you see such an affection, always listen to the heart. Sometimes in younger persons something can be done. Three years ago had a similar case, which, upon treatment with digitalis, iron and strychnia, recovered.

CASE II.—C——; aged 22; brakeman; has just recovered from typhoid fever, but is now under treatment for epilepsy, which he has had for one year previously. Does not know of any cause. Has had but nine attacks. Never has prodromic symptoms of a seizure. In this case can discover no centric or eccentric cause. He is of good habits, has had no injury; stomach and bowels in good order, no spinal irritation, no masturbation, and no overwork, and it is necessary, therefore, in this instance to prescribe empirically. One of the best agents is Pot. Brom. gr. xx t. i. d., and, if taken for one or two years, may eventual in recovery.

CASE III.—X.; aged 22; baker; taken sick last Saturday; has headache; no bleeding from the nose; pulse 98, temp. 103¾°; entered hospital yesterday. May be malarial fever, which is prevalent, or typhoid, because he has pain and gurgling in right iliac region, but he has cough, slight, rusty expectoration and pain in right side of chest. He has a tremulous red tongue, there is no sordes, his abdomen is tympanitic, liver and spleen healthy; and there is tenderness on percussion also in epigastric region. On examination of chest find soronous rales low down behind and on the right, almost crepitant rales, and bronchophony, and there is dullness on percussion. It is a pneumonia, which is now passing into stage of red hepatization. Advise Ammon. Carb. grs. v every two hours, and Pulv. Ipecac Comp. gr. i, when needed for cough.

### FEBRUARY 16, 1881.

CASE I.—Male; aged 23; double uvula; advises cutting the second one off.

CASE II.—No. 1 in report of Jan. 26.; incontinence of urine. The treatment before mentioned was carefully carried out: Tr. gram. gtt. x t. i. d. and Fl. ext. ergot. ʒi at 6 and 12 P. M. It has resulted in perfect recovery.

CASE III.—Male; aged 30; epigastric tumor presumed to be aortic; has been seen by the class earlier in the season; at one time had many symptoms which would point to nickel poisoning. A purring thrill is heard over aorta in the epigastric region; he has been treated with Pot. iodide gr. v and Corros. lub. gr. $\frac{1}{64}$ t. i. d., with the result that the tumor is smaller than when last examined.

CASE IV.—Ch. H.; aged 32; entered hospital day before as an epileptic; a stranger in the city; during preceding night was taken with acute mania, and is now brought in to the amphitheatre in straight-jacket. When epilepsy and mania are associated, a centric origin is indicated. A common centric cause is that of a spiculum of bone pressing upon the brain, a result of fracture of the skull. The only remedy in such cases is trephining. Upon examining the skull of this patient a large indenture is found on the left side of the calvarium. No history of the injury can be obtained. Patient, in his sane moments, tells various stories, that he fell down stairs, that it is an old bullet wound, etc. There can be little doubt that this a case requiring trephining. A hypodermic injection of hyoscyamine gr. $\frac{1}{10}$ the night before had quieted him effectually. Dr. Andrews, of the State Insane Asylum, says, this is of little value when administered by the stomach, but is exceedingly efficient when used subcutaneously. In the hospital it has been used in delirium tremens in $\frac{1}{24}$ gr. doses hypodermically with good effect. This form of epilepsy is called the *grand mal.* There is another form known as the *petit mal*, which is equally important and, indeed, may result in greater epilepsy; its manifestation is slight, it may be but a vacant stare of a few seconds, occurring at any time, and those subject to it are often called absent-minded. It should not be lost sight of. It often eventuates in insanity. As a rule, epilepsy impairs the memory and affects the mind, but sometimes a brilliant intellect may be co-existent with it, as in Julius Cæsar and Napoleon Bonaparte. This should always be held out as a hope to such patients and their friends.

CASE V.—No. 3 of last week. It was diagnosed as an incipient pneumonia, which was correct. The remarkable point in the case is that this patient for three days had a *temperature of 108°* and is nevertheless convalescent.

