# Errata

**Published:** 1881-03

**Authors:** 


### ERRATA.

IN THE MARCH NUMBER.

Page 349, 16th line from above: Pulv. Ipecac Comp. gr. I, read Pulv. Ipecac Comp. gr. V.

12th line from below: Gram read Ferri chlor. and Fl. ext. ergot ℥i read Fl. ext. ergot ʒi.

